# Assessment of environmental pollutants at trace levels using ionic liquids-based liquid-phase microextraction

**DOI:** 10.55730/1300-0527.3479

**Published:** 2022-07-19

**Authors:** Furkan UZCAN, Muhammad Saqaf JAGIRANI, Mustafa SOYLAK

**Affiliations:** 1 Faculty of Sciences, Department of Chemistry, Erciyes University, Kayseri, Turkey; 2 Technology Research and Application Center (ERUTAUM), Erciyes University, Kayseri, Turkey; 3 National Center of Excellence in Analytical Chemistry, University of Sindh, Sindh, Pakistan; 4 Turkish Academy of Sciences (TUBA), Ankara, Turkey

**Keywords:** Sample preparation methods, ionic liquids, liquid-phase microextraction, toxic pollutants

## Abstract

Sample preparation is the crucial and most challenging part of analytical chemistry for the speciation of environmental pollutants’ traces. Along with the development of the sample preparation methods, the ionic liquid-based microextraction technique plays an important role. Due to the unequivocally unique “green” characteristic of ionic liquids (ILs), they owe their tunable properties, such as highly selective and high reaction efficiency, reusability, and good thermal stability, to present advancements in the sample preparation process. The ionic liquid-based microextraction techniques miniaturize the sample preparation process. Liquid phase microextraction intermediate solvents, desorption solvent extractants, and mediators have been used. They are quoting the benefits and limitations of each method. A few essential sample preparation methods covered the microextraction technique. In this context, miniaturized microextraction methods have been developed. They are generally used for their unlimited positive features, including easy, simple, and environmentally friendly; they also extract inorganic and organic species with low-cost instrumentation. This review advances the sample preparation process using ILs-based liquid phase microextraction as an intermediate solvent, extractant desorption, and mediator solvents.

## 1. Introduction

The sample preparation process is an essential part of analytical chemistry; it plays a critical role in qualitative and quantitative analysis and obeys the rules of green analytical chemistry (GAC). A classic analytical process consists of three crucial parts: sampling, preparation of the sample, and analysis. It usually takes 75% of the researcher’s time in the preparation stage [1]. During sample preparation, conventional methods sometimes violate the principle of green analytical chemistry (GAC). The GAC describes the philosophy of the pro-ecological accomplishments in analytical laboratories. The quantitative determination of chemical compounds in trace or ultra-trace analytical samples generally requires an initial step of isolating analytes. It is associated with the performance of the analytical techniques, and in some cases, it is not sensitive enough for rapid identification at such a low concentration [2–11]. Several modern technological approaches have been proposed in this field [12–16]. One such class of compounds called “modern era solvents” is ionic liquids (ILs). ILs have been used as sorption constituents in recent decades. The rapid growth of ILs is related to their unique characteristics, particularly those properties that are essential from an “environmental” point of view, i.e. low flammability, high thermal stability, and negligible vapor pressure due to these unique properties [17, 18].

## 2. Ionic liquids

**Figure 1:**
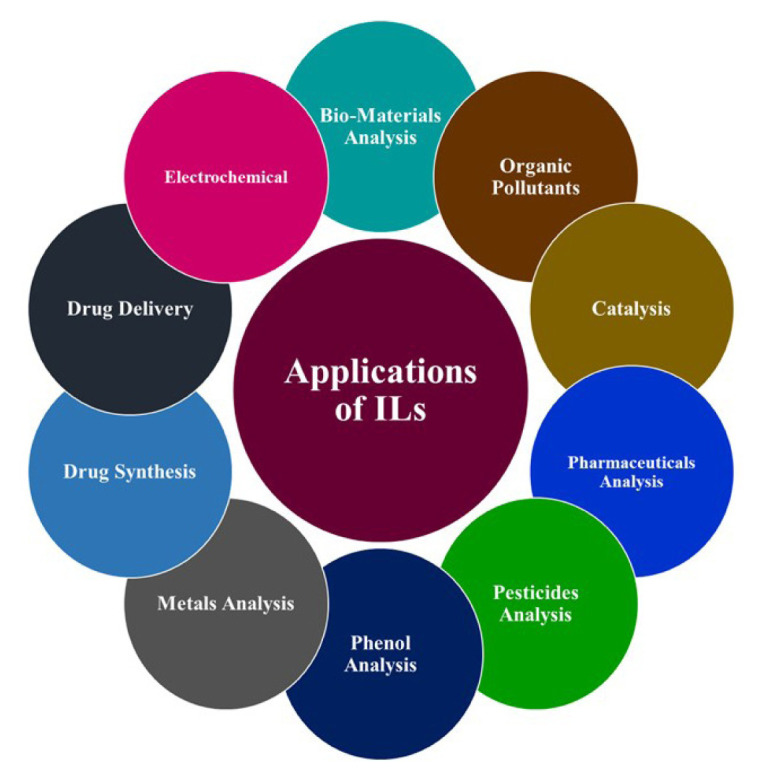
The general applications of ILs.

Ionic liquids (ILs) are a new group of solvents with unique features such as tunable physicochemical properties by the interchanging of cations and anions, slight vapour pressure, and good capability with a wide range of analysts via nonpolar, ionic, or specific interactions that make them an ideal candidate and alternative to traditional organic solvents for the sample preparation process. ILs have recognized physical features that raise more interest in present times. Generally, a predictable, convenient description of an ILs is a salt with a melting temperature of below 100 °C and is formed from inorganic anions and organic cations. In the reported literature, the simple definition of ILs is typically defined as compounds that are entirely composed of ions and have a melting point of less than 100 °C [19]. Different synonyms have been declared for ionic liquids. From that, molten salts are the most familiar term. ILs have broad applications in the ionic species in the molten state [20]. The difference between ionic liquids and molten salts appears to be only a matter of degree. However, the functional distinctions are enough for the liquid salts at room temperature to explain a separately defined role. ILs will typically be viewed as ordinary solvents in studies. Such essential characteristics of ILs are often present in the ion-ion solid interactions that are not found in molten salts at higher temperatures. In a great diversity of consumer and industrial applications, heat-transfer fluids are found. Applications vary from cooling devices at low temperatures to high-temperature processing and storage of solar energy. The most popular heat-transfer fluid is potentially steam [21]. The industrial applications of ILs are aluminum plating, paint additives, hydraulic ILs compressors, batteries, and solar cells [22]. The ILs-based separation and extraction approach is a modern approach that takes the place of volatile organic compounds as an extract [23, 24]. The properties of ILs make them predominantly suitable for solvent extraction, including their combustibility and low volatility, thermal stability, wide liquid range, adjustable functional groups, high conductivity, and a wide range of electrochemical applications [25–28]. The ILs have shown excellent performance in the extraction techniques used in sample preparation and preconcentration of targeted analytes [29–34]. ILs contain low melting point salts. They are generally attained by using large asymmetric cations and faintly coordinating with anions [35]. Researchers pay more attention to ILs due to their tunable features such as high chemical and thermal stability, lower vapor pressure, and an increased temperature range in the liquid form. The branching and length of alkyl chains and anionic precursors can produce “designer solvents” for task-specific applications, such as the extraction of different analytes from real samples [36–38]. The ILs have been significantly used in solvent-based dispersive liquid-liquid microextraction (DLLME) techniques [39]. First, organic salt (ethylammonium nitrate ([EtNH_3_]NO_3_) was discovered in 1914 and found to be liquid at 25 °C with a low melting point. Usually, organic cations include imidazolium, pyrrolidinium, ammonium, pyridinium, inorganic anions, tetrafluoroborate, chloride, bromide, and hexafluorophosphate contain ILs [39–41]. Although several analytical and industrial processes have been applied to the ILs, their usage in wastewater treatment, particularly in eliminating organic pollutants, is limited. Hurley et al. [42] reported ILs using AlCl_3_ and N-alkylpyridine to heat the solids mixture and found a clear, transparent liquid. In particular, ILs have been introduced as an attractive alternative to traditional organic solvents in a wide range of chemical and biological procedures [43]. ILs also extract organic and inorganic toxic pollutants [44, 45]. Over the last few years, the extraction of different metal ions using ionic liquids having appropriate complexing agents, including dithizone [46], and various other organic ligands, has been carried out [47]. Toxic metals have been removed from the aqueous environment by different methods such as flotation, chemical precipitation, adsorption, electrochemical deposition, and ion exchange. These reported methods have some limitations, such as time-consuming, selectivity sensitivity, and costly, negatively impacting the environments [48–51]. Liquid-liquid extraction (LLE) has some limitations, it is time-consuming, requiring a large amount of solvent during the sample preparation and preconcentration, and it has limited applications. Microextraction techniques are ideal candidates for overcome these limitations because they are fast, selective, sensitive, and environmentally friendly methods for sample preparation and preconcentration. Figure 1 shows general applications of ILs.

### 2.1. Advantages of ILs

The key advantages of ILs when used for the SDME are that they permit the application of longer the sampling time and the large volume that has been used, leading to optimized HPLC protocols for sensitive determination [52]. Wang et al. reported a new method, the capillary electrophoresis (CE) hyphenated with the SDME, to extract phenols from the aqueous environment [53]. In this method, approximately 2.40 nL of 1-butyl-3-methylimidazolium hexafluorophosphate ([C_4_MIM][PF_6_]) has been used as an extraction solvent for the online combination of SDME. The EF was obtained up to 107–156, showing a higher sensitivity than the reported methods. The ILs-based SDME have been efficiently used to analyze heavy metals from biological and environmental samples [54–56]. HF-LPME-based ILs have been used in the LPME [57–64]. ILs are nonvolatile and polar. Reported studies also established that an ILs in the pores of the supported membrane could be evacuated, and the supported ILs membrane was moderately stable under the insignificant stirring conditions [65, 66]. Moreover, ILs have a high affinity toward the polar compounds [67] and the ILs membrane could transport some organic compounds selectively [68–72]. Table 1 shows the different applications of ILs based LPME.

### 2.1. Limitation of ILs

ILs can become persistent pollutants that threaten the environment and are cost-effective, making them unsuitable for larger industrial applications such as metal electroplating, electrodeposition, and biocatalyst. As a result, the usage and cost issues have been the primary challenges in traditional ILs applications. A variety of issues, including toxicity and availability, will limit their practical use for larger-scale applications of other metals and biomaterials. Even though many recipes for the synthesis of traditional ILs have been published, not all applied research laboratories have the expertise, work practices, and equipment required to complete synthesis due to complicated synthetic processes. Furthermore, it is frequently challenging to prepare pure, dried traditional ILs or carry out postsynthesis purification steps [73].

On the other hand, the commercial availability of some traditional ILs has limited small volumes, or the cost of many liquids remains prohibitively expensive for applied engineering research. The high cost of synthesis, incompatibility with GC due to low volatility, and toxic effects. In general, ionic liquid research will continue to develop as the need for green analytical techniques becomes a priority in sample preparation [74].

## 3. ILs-based microextraction process

The advancement and development of novel sustainable analytical processes are crucial for GAC [75–82]. The application of state-of-the-art solvents, such as ILs, hyphenated with microextraction methods could be an outstanding approach for environmentally friendly sample preparation compared to classical methods. Some of the GAC, such as waste generation or minimal, use of safer solvents, and improvement of miniaturized approaches are fulfilled by introducing ILs and microextraction in the analytical approach. ILs are widely used in sample preparation methodologies and are commonly used in the routine analysis in laboratories to extract and determine analytes at the trace level. Several publications have been reported on the ILs based microextraction method. Different microextraction-based methods have been reported, such as solid-phase microextraction (SPME) and liquid-phase microextraction (LPME) [19, 83–85]. LPME appeared from LLE, one of the most common extraction techniques for inorganic and organic sample preparation, preconcentration, and analysis [86]. ILs have been offered as extraction solvents and ion-pairing agents along with the liquid-liquid microextraction (LLME) methods for the extraction of metals and organic compounds with a low limit of detection (LOD), the sensitivity and selectivity of incomplete analysis and speciation of some metals, and organic compounds [87–89]. Different ILs based LLME approaches have been proposed, such as single-drop microextraction (SDME) and dispersive LLME (DLLME) vortex-assisted liquid-liquid microextraction (VA-LLME) [90–94].

### 3.1. ILs-based LPME

**Figure 2:**
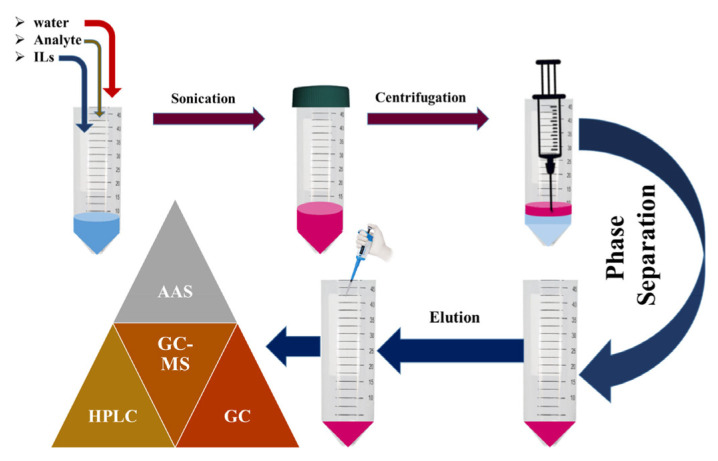
represents the working mechanism of ILs-based LPME.

LPME has recently established sample preparation and analytical techniques using negligible amounts of solvent. This technique is fast, easy, highly selective and sensitive, and environmentally friendly, and a minimal amount of organic solvents has been used. The working protocols are associated with the isolation, preconcentration, sample preparation, and introduction in a single step. In ILs-based LPME sample preparation and preconcentration, a small amount of solvents with hydrophobic dissolvent in aqueous media (aqueous sample/donor phase) have been used to extract the targeted analyte [95, 96]. In the advancement of analytical chemistry, the ILs-based LPME process opens a new door in sample preparation due to miniaturization, automation, and facilitation. The LPME miniaturized the extraction processes and analysis of organic and inorganic compounds [75, 97–103]. The ILs-based LPME techniques have been generally hyphenated with different analytical methods, such as atomic fluorescence spectrometry (AFS), atomic absorption spectroscopy (AAS), and inductively coupled plasma spectrometry (ICP), to quantify the ultra-trace level of analytes from food, biological, and environmental samples [83, 104–116]. Metals have been directly analyzed using the ILs phase with a small amount of 10–50 μL of organic solvents (ethanol or methanol). While during the extraction of metals, the ILs often reveal high selectivity and high efficiency, metal ions usually separate deeply based upon the kinds of ILs, the metal ions ligands [83, 117–120], so different processes have been developed for the separation of analytes from water samples [75]. The extraction and precontraction of metals using crown ethers result in crown ether complexes showing high hydrophobicity when retentive to present electric charge [121, 122]. The neutral complex ligands have been used for the extraction of metals [123, 124] and are widely used for the elimination of different metals, such as aluminum [125, 126], mercury [127], or nickel; for the metal extraction of the ILs based on the cationic replaceable group in their structure [128–130]. The ILs-based LPME has been used in the different extraction techniques. They are classified into three different methods such as IL-based single-drop microextraction (IL-SDME), IL dispersive liquid-liquid microextraction (IL-DLLME), and IL-hollow-fiber LPME (IL-HF-LPME). A number of modifications have also been introduced for these methods, which exhibit the versatility of the technique [131]. Figure 2 represents the general working mechanism of ILs-based LPME process.

#### 3.1.1. ILs-based SDME

Dasgupta’s [131] research group first proposed a method in 1995 containing a liquid droplet as a gas sampling edge to extract substances, such as sulfur dioxide and ammonia, from the air [131, 132]. Jeannot and Cantwell reported solvent-based microextraction into a single drop in 1996 to determine the organic compounds [133]. The authors used an 8 μL drop of organic solvent (n-octane) and thought about hollowing out the Teflon rod occupied in the water sample to eliminate 4-methyl acetophenone [134]. He and Lee present a standard micro-syringe for single drop microextraction [135]. In current practice, solvent-based microextraction into a single drop is commonly known as SDME. There are different formats such as (continuous (cycle) flow, IL-CFME, two-phase direct immersion, IL-DI-SDME), and three-phase (headspace, IL-HS-SDME) have been used with ILs as the extractant for the sample preparation before the detection of metal ions. SDME method requires a very small volume (1– 3 μL) of solvent and is economically beneficial, fast, and easy to operate. It can be applied with a simple device, e.g., a traditional micro-syringe [95, 134, 136]. In 2003, Lin et al. reported the application of ILs (1-octyl-3-methylimidazolium hexafluorophosphate) as an extraction solvent in the SDME for the extraction objective. The authors applied SDME in both HS and DI modes to extract and preconcentrate model compounds. In DI mode, higher enrichment factors (EFs) were attained for the polycyclic aromatic hydrocarbons. However, the EF reported for HS-SDME of naphthalene was around three times larger than DI-SDME. For this reason, they verified that the HS-SDME was long-lasting compared to the DI-SDME when unstable analytes had been extracted. The advancement in the DI-SDME method uses ILs for extraction and preconcentration of metals [52].

#### 3.1.2. ILs-based DLLME

DLLME technique was firstly introduced by Rezaee et al., 2006 [137]. The fundamental basis of DLLME includes the addition of hydrophobic elimination solvent and dispersive solvent to the water sample. This is the principle for the development of a cloudy solution. Over centrifugation, a two-phasic scheme is recognized. DLLME offers high preconcentration factors because of the dispersive solvent that results in the establishment of micro-droplets that raise the interaction area of the extraction solvent. Zhou et al. reported temperature-controlled IL-DLLME to eliminate pyrethroid pesticides from aqueous solutions [138]. The temperature was optimized up to 70 °C for a complete dissolution of [C_6_MIM][PF_6_] ILs to boost the separation of the analyte into the ILs phase. The ILs were then centrifuged or cooled for the phase separation of the sample. Zhou et al. proposed another method without using heat, just using ultrasonication in order to improve the dissolution [139]. Liu et al. used acetone and methanol with [C_6_MIM][PF_6_] to prepare ILs as extraction solvents, and acetonitrile was used as a dispersive solvent to extract different heterocyclic insecticides, such as chlorfenapyr, fipronil, hexythiazox, and ibuprofen. Valcarcel et al. proposed a new DLLME-based method that used a syringe to prevent centrifugation [140–142].

#### 3.1.3. ILs-based HF-LPME

HF-LPME could be performed in two-phase and three-phase modes. In the two-phase mode, the aqueous immiscible organic solvent is used to fill both walls’ pores and the hollow-fiber lumen. This mode has been used for the elimination of hydrophobic analytes. The targeted analytes are eliminated from the water samples in the three methods through the water-immiscible organic solvent immobilized in the hollow fiber pores into the aqueous acceptor phase present in the hollow fiber lumen. In this circumstance, the analyte must exist in two forms: in a nonionic form on the sample side to be eliminated into the membrane, and in an ionic arrangement on the acceptor side to be irreversibly confined. This is commonly attained by pH adjustments in the two aqueous phases. Therefore, the process is mainly well-suited for ionizable analytes such as weak alkaline and acidic media. The sample volume in HF-LPME ranges between several hundred μL and more than 1 L, whereas the volume of acceptor solution in most cases ranges up to 2–25 μL [143–146].

## 4. Applications of ILs based microextraction

ILs-based SDME has various biological and environmental applications [53, 147–156]. In 2003 Liu et al. firstly reported the use of ILs in the SDME [147]. Three ILs containing the 1-butyl-3-methylimidazolium hexafluorophosphate ([C_4_MIM] [PF_6_]), [PF_6_^−^] anion, [C_8_MIM] [PF_6_], and 1-hexyl-3-methylimidazolium hexafluorophosphate ([C_6_MIM] [PF_6_]) have been used as the extraction phase hyphenated with high performance liquid chromatograph (HPLC) to the determination of polycyclic aromatic hydrocarbons (PAHs) from the water samples. Compared to 1-octanol, larger micro-droplets were formed using [C_8_MIM] [PF_6_], subsequent in different orders of magnitude increase in the EF. Future, a 10 μL droplet of ILs was occupied in the sample solution, resulting in the percentage recoveries between the 90% and 113% of two main 4-nonylphenol and 4-tert-octylphenol (alkylphenols) in the aqueous environment.

## 5. Conclusion and future directions

The novel and tunable chemical and physical properties of ILs enable the preparation and design of highly selective ILs for the selective and sensitive analysis of targeted analytes. The researchers have been paying more attention to the design, synthesis, and cost-effective environmentally friendly method to prepare ILs. The preparation design of cost-effective and more functional ILs will be a goal in the ILs-based LPME process. As mentioned above, one of the most challenging applications of ILs is applied in the microextraction methods as mediators, intermediate solvent and extractants, and desorption solvent. The ILs-based microextraction methods have promising properties and advantages that are not only used in microextraction techniques but will be enhanced by the separation techniques such as liquid and electrophoresis or gas chromatography will expand their applications. The applications of ILs in the LPME of the toxic environmental samples have had significant development during the last decades, from their use as cosolvent to their tremendous role as solvents and reagents. New designs have led to a wide range of specific applications of ionic liquids and more selective approaches. That increases the effectiveness of traditional extractants in complex matrices like environmental and food. These solvents have been integrated into all LPME approaches, although DLLME and HF*LPME have seen remarkable advancement. ILs have enabled new modes of operation in DLLME of environmental pollutants such as in situ, TC-DLLME, USA-DLLME, AA-DLLME, and EA-DLLME. Task-specific ILs (TSILs) in HF-LPME have been a reliable method for metal speciation from complicated matrices. ILs can widen the use of LPME to extract polar analytes with a low detection limit. New ILs-based ion-pairing compounds will likely soon be mercantile existing to assist the detection of analytes using ESI-MS. Overall, ILs have come up as versatile and unique solvent materials with many advantages and will continue to gain more attention from the mass spectroscopy and separation science societies. Finally, improvements in instrumental procedures have been made to adapt to the ILs matrix, including the invention of lab-on-a-disk metal determination. In the future, LPME developments include the focused development of TSILs with increased selectivity for metallic species and deep eutectic solvents based-liquid phase micro extraction (DES-LPME). Finally, research on optimizing instrumental determination and incorporating microfluidic technologies may enable the wider use of IL-LPME in metals.

## Figures and Tables

**Table:** Representation of the different applications of ILs based microextraction.

Ionic liquid(s)	Analyte(s)	Sample	Technique	Instrument	LOD (all units are μg/L unless otherwise stated)	Ref.
1-ethyl-3-methylimidazolium hexafuorophosphate	Pioglitazone	Drug samples	Dispersive liquid–liquid microextraction	HPLC	10	[157]
1-octyl-3-methylimidazolium chloride	Te and Se	Environmental samples	In-situ solvent formation microextraction	HG-AFS	0.0026–0.0032	[158]
1-hexyl-3-methylimidazolium chloride, Lithium bis(trifluoromethyl) sulfonylimide	Chlorobenzenes	Environmental samples	Dispersive liquid-phase microextraction	GC-MS	0.0084–0.252 ng/L	[159]
1-butoxy-3-ethoxy-2-ethyl-imidazolium bis(trifluoromethane)sulfonimide	Cannabidiol	Natural cosmetics	Liquid-phase microextraction	HPLC-UV	-	[160]
1-butyl-3-methylimidazolium chloride	Carbamate pesticides	Packed fruit juice samples	Dispersive liquid-phase microextraction	HPLC-DAD	0.4–3.9	[161]
1-butyl-3-methylimidazolium hexafuorophosphate, 1-hexyl-3-methylimidazolium hexafuorophosphate, 1-octyl-3-methylimidazolium hexafuorophosphate	Triazole fungicides	Water samples	Dispersive liquid-phase microextraction	HPLC-DAD	0.19–0.55	[162]
1-butyl-3-methylimidazolium methylsulfate	Glucocorticoids	Water samples	Liquid-phase microextraction	HPLC-MS/MS	0.0128–0.0470	[163]
1-Ethyl-3-methylimidazolium tetrafluoroborate, 1-propyl-3-methylimida-zolium tetrafluoroborate, 1-buhyl-3-methylimidazolium tetrafluoroborate	Phenols	Environmental water samples	In-tube liquid-phase microextraction	CE	1.0–5.0	[164]
Trihexyl(tetradecyl)phosphonium bis[(2,4,4-tri-methyl)pentyl]phosphinate	Heavy Metals	Water samples	Dispersive liquid–liquid microextraction	LC-UV	0.02–0.03	[165]
1-hexyl-3-methylimidazolium hexafluorophosphate	Naphthoquinones	Zicao	Magnetized stirring bar liquid-phase microextraction	HPLC	80–120	[166]
1-silicyl-3-benzylimidazolehexafluorophosphate	Polycyclic aromatic hydrocarbons	Water samples	Headspace liquid-phase microextraction	HPLC-Flu	0.003–0.015	[167]
1-butyl-3-methylimidazoliumhexafluorophosphate	Thiabendazole	Food samples	Liquid-phase microextraction	UV-Vis	0.1–0.24	[168]
Tricapryl-methylammonium chloride	Sb and Sn	Beverage samples	Dispersive liquid–liquid microextraction	ICP-OES	0.0025–0.0012	[169]
1-butyl-3-methylimidazolium bromide	Brazilin and Protosappanin B	*Caesalpinia sappan*	Dispersive liquid-phase microextraction	HPLC-UV	–	[170]
1-Butyl-3-methyl imidazolium chloride, Sodium hexafluorophosphate	Ni, Cu, and Zn	Wastewater and alloy samples	Dispersive liquid phase microextraction	FAAS	0.71–0.93	[171]
1-butyl-3-methy-limidazolium hexafluorophosphate	Phthalate esters	Tea samples	Hollow fibre liquid phase microextraction	HPLC-DAD	0.67–1.73	[172]
1-butyl3-methylimidazolium hexafluorophosphate	Diclofenac and mefenamic acid	Urine samples	Liquid-phase microextraction	HPLC-UV	20–30	[173]
1-Hexyl-3-methylimidazolium hexafluorophosphate	Mn	Environmental water samples	Dispersive liquid phase microextraction	ETAAS	0.023	[174]
1-hexyl-3-methylimidazolium hexafluorophosphate, sodium hexafluorophosphate	Co and Ni	Biological samples	Liquid-phase microextraction	FAAS	0.03–0.09	[175]
1-hexyl-3-methylimidazolium bromide	Aconitum alkaloids	*Aconitum carmichaeli*	In situ liquid–liquid microextraction	HPLC	0.048–0.082	[176]
tributyldodecylphosphonium tetrafluoroborate	Phthalate esters	Environmental samples	Liquid-phase microextraction	HPLC	0.27–2.36	[177]
tributyldodecylphosphonium hexafluorophosphate	Pyrethroid insecticides	Water samples	Liquid-phase microextraction	HPLC	0.71–1.54	[177]
1-hexyl-3-methylimidazolium hexafluorophosphate	Ferro and ferric	Environmental water samples	Hollow fiber liquid-phase microextraction	FAAS	0.4–0.6	[178]
1-butyl-3-methylimidazolium tetrachloroferrate	Triazine herbicides	Oil Seeds	Dispersive liquid–liquid microextraction	UFLC-UV	1.20–2.72 ng/g	[179]
1-hydroxyethyl-3-methylimidazolium bis(trifluoromethanesulfonyl)imide	Three estrogens and bisphenol A	Environmental water samples	Dispersive liquid phase microextraction	HPLC-UV	1.7–3.4	[180]
1-alkyl-3-methylimidazolium bromide	Synthetic food colourants	Food samples	Liquid–liquid microextraction	HPLC-UV	0.051–0.074	[181]
1-Hexyl-3-methylimidazolium hexafluorophosphate	Carvedilol	Biological samples	Liquid-phase microextraction	Spectrofluorometer	1.7	[182]
1-butyl-3-methylimidazolium bis (trifluoromethyl) imide	Methamphetamine	Urine samples	Dispersive liquid phase microextraction	HPLC	10	[183]
1-butyl-3-methylimidazolium hexafluorophosphate, 1-octyl-3-methylimidazolium hexafluorophosphate, 1-octyl-3-methylimidazolium tetrafluoroborate, 1-hexyl-3-methylimidazolium hexafluorophosphate, 1-hexyl-3-methylimidazolium hexafluorophosphate	Hg	Water samples	Hollow fiber liquid phase microextraction	UV-Vis	0.2	[184]
1-octyl-3-methylimidazolium hexafluorophosphate	Sulfonamides	Butter samples	Liquid-phase microextraction	HPLC	1.20–2.17 ug/kg	[185]
trihexyl(tetradecyl)phosphonium hexafluorophosphate	Benzoylurea insecticide	Fruit juice	Liquid-phase microextraction	HPLC-VWD	0.03–0.28	[186]
1-butyl-3-methylimidazolium hexafluorophosphate	Cd	Various Samples	Hollow fiber liquid phase microextraction	GF-AAS	0.00012	[187]
1-Hexyl-3-methylimidazolium hexafluorophosphate	Anethole, estragole, and para-anisaldehyde	Plant extracts and human urine	Dispersive liquid phase microextraction	HPLC	47–60	[188]
1-Octyl-3-methylimidazoliumhexafluorophosphate	Triazine herbicides	Water samples	Dispersive liquid phase microextraction	HPLC	0.05–0.06	[189]
1-butyl-3-methylimidazolium hexafluorophosphate, 1-hexyl-3-methyl-imidazolium hexafluorophosphate	Bisphenol A and bisphenol AF	Vinegar samples	Dispersive liquid Phase microextraction	HPLC	0.15–0.38	[190]
1-butyl-3-methylimidazolium hexafluorophosphate	Cd	Water samples	Dispersive liquid phase microextraction	FAAS	0.62	[191]
1-Octyl-3-methylimidazolium hexafluorophosphate	Bisphenol A, 17-β-estradiol, estrone and diethylstilbestrol	Water samples	Hollow-fiber-supported liquid-phase microextraction	HPLC	0.03–0.10	[192]
1-butyl-3-methylimidazolium hexafluorophosphate	Cd	Biological samples	Dispersive liquid phase microextraction	FAAS	0.05	[193]
1-Octyl-3-methylimidazolium tetrafluoroborate	Synthetic food colourants	Various samples	Dispersive liquid phase microextraction	HPLC	0.015–0.32	[194]
1-butyl-3-methylimidazolium hexafluorophosphate	Pb	Biological Samples	Liquid phase microextraction	FAAS	5.8	[195]
1-Hexyl-3-methylimidazolium hexafluorophosphate	Bisphenol A and 4-Nonylphenol	Water samples	Dispersive liquid phase microextraction	HPLC	0.055–0.76	[196]
1-octyl-3-methylimidazolium hexafluorophosphate	Fungicides	Water samples	Dispersive liquid phase microextraction	HPLC-UV	0.32–0.79	[197]
1-octyl-3-methylimidazolium hexafluorophosphate, 1-octyl-3-methylimidazolium bis (trifluoromethanesulfonyl) imide, 1-octyl-3-methylimidazolium tetrafluoroborate	Aromatic Amines	Water samples	Dispersive liquid phase microextraction	HPLC	0.39–0.63	[198]
1-butyl-3-methylimidazolium hexafluorophosphate	Dichlorvos	Environmental samples	Dispersive liquid phase microextraction	HPLC	0.2	[199]
1-Butyl-3-methylimidazolium hexafluorophosphate	Pb	Blood samples	Dispersive liquid phase microextraction	FAAS	0.13	[72]
1-hexyl-3-methylimidazolium hexafluorophosphate	Co	Nutritional supplements	Dispersive liquid phase microextraction	ETAAS	0.0054	[200]
1-octyl-3-methylimidazolium hexafluorophosphate	Sulfonamides	Environmental water samples	Single-drop liquid-phase microextraction	HPLC	0.5–1	[201]
1-butyl-3-methylimidazolium chloride	Polycyclic aromatic hydrocarbons	Water samples	Hollow-fiber protected liquid phase microextraction	HPLC	0.25	[64]
Tetradecyl(trihexyl)phosphonium chloride	Tl	Water samples	Dispersive liquid–liquid microextraction	ETAAS	0.0033	[202]
1-butyl-3-methy-limidazoliumhexafluorophosphate	Benzene, toluene, ethylbenzene and xylenes	Water samples	Hollow fiber supported liquid-phase microextraction	GC-FID	2.2–4.0	[60]
1-hexyl-3-methylimidazolium hexaflorophosphate	Ag	Various samples	Dispersive liquid phase microextraction	GF-AAS	0.0052	[203]
1-octyl-3-methylimidazolium hexafluorophosphate	Bisphenol A, 4-n-nonylphenol, and 4-tert-octylphenol	Water samples	Dispersive liquid phase microextraction	HPLC-FLD	0.23–0.48	[204]
1-octyl-3-methylimidazolium hexafluorophosphate	Phthalate esters and pyrethroid insecticides	Water samples	Dispersive liquid phase microextraction	HPLC	0.23–0.47	[205]
1-butyl-3-methylimidazolium hexafluorophosphate	Phenols	Seawater samples	Three-phase liquid–liquid-liquid solvent bar microextraction	HPLC-UV	0.01–0.1	[206]
1-hexyl-3-methylimidazolium hexafluorophosphate	Fungicides	Wine samples	Dispersive liquid phase microextraction	HPLC	2.8–16.8	[207]
1-hexyl-3-methylimidazolium hexafluorophosphate	Carbamate pesticides	Water samples	Dispersive liquid phase microextraction	HPLC-UV	0.45–1.40	[208]
1-hexyl-3-methylimidazolium hexafluorophosphate	Tetrabromobisphenol A	Environmental water samples	Dispersive liquid-phase microextraction	HPLC–ESI-MS–MS	0.06	[209]
1-octyl-3-methylimidazolium hexafluorophosphate	Phenols	Water samples	Dispersive liquid-phase microextraction	HPLC	0.27–0.68	[210]
1-Hexyl-3-methylimidazolium hexafluorophosphate	Hexabromocyclododecane diastereomers	Water samples	Dispersive liquid-phase microextraction	RRLC–ESI-MS–MS	0.1	[211]
1-hexyl-3-methylimidazolium hexafluorophosphate	Benzoylureas pesticides	Environmental water samples	Dispersive liquid-phase microextraction	HPLC	0.21–0.45	[212]
1-octyl-3-methylimidazolium hexafluorophosphate	Triclosan, triclocarban and methyl-triclosan	Water samples	Dispersive liquid-phase microextraction	UHPLC-TUV	0.00115–0.00533	[213]
1-butyl-3-methylimidazolium hexafluorophosphate	Chlorobenzenes	Water samples	Dispersive liquid-phase microextraction	HPLC-DAD	0.05–0.1	[137]
1-hexyl-3-methylimidazolium hexafluorophosphate	Herbicides	Water samples	Dispersive liquid-phase microextraction	HPLC-DAD	0.46– 0.89	[214]
1-Hexyl-3-methylimidazoliumhexafluorophosphate	Pb	Water samples	Liquid-phase microextraction	FAAS	9.5	[215]
1-hexyl-3-methylimidazolium hexafluorophosphate	Chlorotoluron, diethofencarb and chlorbenzuron	Water samples	Dispersive liquid-phase microextraction	HPLC-UV	0.04–0.43	[216]
1-butyl-3-methyl-imidazolium hexafluorophosphate	Phenothiazine derivatives	Urine samples	Dynamic liquid-phase microextraction	LC-UV	21–60	[217]
1-Hexyl-3-methylimidazolium hexafluorophosphate	Organophosphorus pesticides	Water samples	Dispersive liquid-phase microextraction	HPLC-UV	0.17–0.29	[218]
1-hexyl-3-methylimidazoliumhexafluorophosphate	Benzophenone-3	Urine samples	Single-drop microextraction	LC	1.3	[219]
1-butyl-3-methylimidazolium hexafluorophosphate	Phenols	Environmental water samples	Headspace liquid-phase microextraction	HPLC	0.3–0.5	[220]
1-butyl-3-methylimidazolium hexafluorophosphate	Chlorinated anilines	Environmental water samples	Headspace liquid-phase microextraction	HPLC	0.5–1.0	[149]
1-octyl-3-methylimiazolium hexafluorophosphate	Formaldehyde	Shiitake mushroom	Liquid-phase microextraction	HPLC	5.0	[148]

## Abbreviations

AAS: Atomic Absorption Spectroscopy
AFS: Atomic Fluorescence Spectrometry
AlCl_3_: Aluminum Trichloride
DI-SDME: Direct Immersion Single Drop Microextraction
DLLME: Dispersive Liquid-Liquid Microextraction
EFs: Enrichment Factors
GAC: Green Analytical Chemistry
GC: Gas Chromatography
HF-LPME: Hollow Fiber-based Liquid-Phase Microextraction
HPLC: High-Performance Liquid Chromatography
ICP: Inductively Coupled Plasma Spectrometry
IL-DLLME: Liquid-Liquid Microextraction
IL-HF-LPME: IL-hollow-fiber LPME
ILs: Ionic Liquids
IL-SDME: IL-based Single-Drop Microextraction
LLME: Liquid-Liquid Microextraction
LOD: Limit of Detection
LPME: Liquid Phase Microextraction
PAHs: Aromatic Hydrocarbons
SDME: Single Drop Microextraction
SPME: Solid-Phase Microextraction
TSILs: Task-Specific ILs
VA-LLME: Vortex-Assisted Liquid-Liquid Microextraction
